# Enhancing Whole Phage Therapy and Their Derived Antimicrobial Enzymes through Complex Formulation

**DOI:** 10.3390/ph11020034

**Published:** 2018-04-19

**Authors:** Callum J. Cooper, Shazeeda Koonjan, Anders S. Nilsson

**Affiliations:** Department of Molecular Biosciences, The Wenner-Gren Institute, Stockholm University, SE-10691 Stockholm, Sweden; shazeeda.koonjan@su.se

**Keywords:** bacteriophage, pharmacology, synergy, formulation, combination therapy, product development

## Abstract

The resurgence of research into phage biology and therapy is, in part, due to the increasing need for novel agents to treat multidrug-resistant infections. Despite a long clinical history in Eastern Europe and initial success within the food industry, commercialized phage products have yet to enter other sectors. This relative lack of success is, in part, due to the inherent biological limitations of whole phages. These include (but are not limited to) reaching target sites at sufficiently high concentrations to establish an infection which produces enough progeny phages to reduce the bacterial population in a clinically meaningful manner and the limited host range of some phages. Conversely, parallels can be drawn between antimicrobial enzymes derived from phages and conventional antibiotics. In the current article the biological limitations of whole phage-based therapeutics and their derived antimicrobial enzymes will be discussed. In addition, the ability of more complex formulations to address these issues, in the context of medical and non-medical applications, will also be included.

## 1. Introduction

Renewed interest in the clinical and non-clinical application of phages and their derived antimicrobial enzymes has been stimulated by the need for new types of antibacterial agents to combat the ongoing problem of antibiotic resistance [1,2,3,4]. Although phages are still often compared to antibiotics, it is well established that they possess both advantages and disadvantages as therapeutic agents. In general, it is accepted that antibiotic treatments result in higher levels of collateral damage to pre-existing microflora due to their non-specific activities [5,6], while phage treatment minimizes this damage [7,8,9]. Therapy based upon the application of whole phages has traditionally been utilized in Eastern Europe to treat a variety of different infections ranging from diabetic foot ulcers to stomach complaints. This type of treatment takes advantage of the lytic replication cycle of wholly-virulent bacteriophages [10,11].

A limited number of whole phage-based products have found success as food additives within Western markets and phage-derived enzymes show promising results, but they are yet to enter the mainstream as human or veterinary therapeutics (Table 1) [12,13,14,15]. There are many obstacles that need to be solved before phages can reach clinical settings and be widely applied in therapy. Many of these issues are associated with the nature and variation of phages, which influences everything from isolation and purification to potential formulation strategies. Each phage possesses unique properties that may or may not be desirable for targeted therapy, e.g., narrow host ranges, pharmacological criteria (burst sizes and latent period), and the ability to maintain bacterial killing in adverse conditions. There are also a number of factors regarding the bacterial host that must be considered, such as the number of targeted bacterial cells, their metabolic state, and the presence of phage defense mechanisms [16,17,18,19]. The vast complexity when these, and other factors, are combined makes it difficult to construct a universal phage formulation for a particular condition that can provide optimal phage efficacy while minimizing deleterious effects. These effects range from competition between phages within a cocktail to clearance by the immune system [20,21,22,23].

Increased levels of knowledge about phage genomes and their function within lytic replication has led to the identification of a number of phage derived proteins which have been shown to be powerful antibacterial agents in their own right [24,25,26,27,28]. These proteins are often produced during the latter stages of the phage replication cycle to aid in the release of newly-assembled phages from the bacterial host and have predominantly focused on endolysins [29,30]. However, it should be noted that other phage derived antimicrobial enzymes, including holins and spanins, have also been investigated [24,31].

Adjuncts and excipients are components of pharmaceutical preparations that can increase the activity of the main active ingredient when administered at the same time (e.g., the combination of caffeine and paracetamol) which play an important role in pharmaceutical formulations [32,33]. In normal pharmaceuticals, these additional components play important roles within the final formulations, ranging from increasing the shelf life of finished products to serving as binding agents, such as alginates, which can hold finished formulations together to produce pills [34,35]. The selection of appropriate adjunct compounds for each formulation is dependent on the nature of the therapy to be developed and is thought to reduce the failure rate in novel drug development [36]. As there have been reports of clinical successes of phage suspensions that presumably do not contain adjuncts, limited attention has been given to the development of more complex phage-based formulations that combine phages and additional pharmaceutical components. For therapies based upon whole phages or their derived antimicrobial enzymes, the formulation of biologically-active components with additional adjuncts may address a number of these factors.

The current review seeks to investigate the general issues associated with whole phage and phage derived antimicrobial enzymes (in particular endolysins as these are the most widely studied) and how they could be incorporated with compatible adjuncts for clinical and non-clinical applications. Areas in which complex formulations could potentially enhance the overall efficacy of therapies will also be highlighted. Although engineered phages and antimicrobial enzymes are also being investigated, they will not form part of the current review.

## 2. Compound Selection and Pre-Formulation Testing

In the development of pharmaceutical adjuncts and novel biocides, clearly established protocols and legislation exist for determining safety and efficacy, both as individual compounds and also when part of formulations, with requirements that vary by application (Table 2) [50]. For phage-derived antimicrobial enzymes, the activity and host range could be easily assessed using pre-existing antibiotic susceptibility testing methods [51,52]. However, there are currently multiple methods that can be used when determining the activity of single whole phages [53,54,55,56]. Although these methods are based upon the ability of the phage to produce plaques in a bacterial lawn, they have been shown to produce varying results [56], and it is widely accepted that only purely-lytic phages should be utilized and that their inherent characteristics (e.g., burst size, host range) should be considered for therapy [57,58], As such, it is essential that rapid, easy to use, quantitative assays are developed which can differentiate between multiple phages.

Once the initial characterization of the phage or antimicrobial enzyme had been performed, and prior to assessing the overall effects of a finished formulation, it would be necessary to know the impact of any potential adverse or additive effects that adjunct compounds provide to the phages or the derived antimicrobial enzymes themselves, as well as the impact upon the bacterial host [59,60]. Perhaps the most obvious concern would be a decrease in the stability or activity of the phages or their derived antimicrobial enzymes. In the case of whole phages, this is likely to arise as the result of broad-spectrum antimicrobial activity which may potentially inactivate the phage along with the bacterial host [59,60,61], while in the case of phage-derived antimicrobial enzymes, this could be achieved through the modification of protein domains as the result of residue oxidation [62]. Therefore, in order to counteract these potentially harmful effects, it would be necessary to optimize the concentrations of these compounds in order to minimize the damage that they could cause to the active component without completely losing any beneficial effects they provide, using standardized test regimes, such as fractional inhibitory concentration (FIC) or checkerboard testing [63,64].

It is also necessary to consider the solubility of both the adjuncts and the phage/enzymes during the initial selection process. Unlike antibiotics, whose concentrations, in many cases, can be increased to levels that are toxic to humans, it is possible that phages will possess an upper threshold of what can be held in suspension, approximately 10^14^ PFU/mL for a small phage, such as T7, due to the differences between their sizes [65]. Such an upper threshold should limit the maximum concentration of each individual component within a cocktail. The limitation on the upper concentration threshold could result in an overall decrease in the level of activity. However, commercially available whole phage products can be routinely produced at titers ≥10^10^ PFU/mL and are used at concentrations of approximately 10^9^ PFU/mL [66]. If necessary, solubility could theoretically be increased through the addition of solubilizing agents, such as polysorbate 80, dimethyl sulfoxide (DMSO), or glycerin [67], it should be noted that some solubilizing agents, such as DMSO, can be antimicrobial at higher concentrations.

## 3. Increasing Antibacterial Activity and Host Range

Among the key double-edged advantages of therapies (based upon whole phages or their derived antimicrobial enzymes compared to conventional antibiotics) is that their specific activity minimizes the collateral damage to commensal microflora at the same time as limiting the host range of each therapy. When designing whole-phage-based therapies, multiple phages are combined into cocktails, with the selection of phages with optimal characteristics of key importance. However, it is often assumed that the best criteria for phage activity are dependent on *in vitro* properties and independent of the type of application [57,58,68,72]. This could lead to the exclusion of some phages which may better suit a particular application. For example, if one was to attempt to reduce the risk of contracting a particular disease in a cattle herd (prevention therapy), such as mastitis, then a phage cocktail which possesses a broad host range, greater environmental stability, and longer lasting lytic activity would prove more useful than a cocktail which targets a limited number of hosts and is susceptible to environmental conditions. Conversely, for active infections against a single characterized bacterium, faster-acting phages with large burst sizes would be more useful (intervention therapy). In the sections below, combinations of phages, their derived antimicrobial enzymes, and additional compounds will be discussed.

### 3.1. Phage Cocktails

Perhaps the most routinely used and well-studied form of formulation, phage cocktails are comprised of multiple phages on the basis of desirable *in vitro* characteristics (e.g., activity and/or host range) in order to enhance the host range and potentiate overall activity when compared to the individual components [73,74,75,76]. A summary of *in vivo* cocktail testing can be found in Table 3. Such combinations possess significantly more complex dynamics compared to individual phages have been reviewed in detail elsewhere and are generally shown to increase antibacterial activity compared to individual phages [76,77,78]. Although phage cocktails are well studied, there are a number of areas in which additional research could be focused on (e.g., increasing phage host range, phage pharmacology, host immune response, etc. [79]), which would provide additional information for the selection of phages in cocktails.

Some of the complexities of active treatment (interventionist) phage cocktail dynamics involve the ability of a single phages to outcompete the other phage components of the cocktail via competition for bacterial binding sites and the loss of phage activity from superinfection exclusion, to lower-risk outcomes which may include the inability to infect resistant bacteria compared to a single phage [18,19,76,78]. While competition for binding sites is perhaps the most obvious example of this, competition could also arise from differences in the replication dynamics of the phage in which one phage possesses a shorter latent period (the time between infection and bacterial lysis) compared to other phages in the cocktail. This competition could, thus, decrease the overall efficacy of the treatment through the faster propagation one of them gets. Indeed, within the published literature, the change in the titer of individual phage components is often not studied and, instead, relies upon the reporting of the total phage content [80,81,82]. Competition between phages for bacterial binding sites could also potentially have a deleterious effect on phage activities in cocktails where phages are applied simultaneously (immediate release), in the absence of any other compounds or formulation and, as such, it would be advantageous to select phages which target different bacterial receptors. Although there are a number of well-characterized examples of surface receptors for phage binding [83,84,85], they are often overlooked when selecting phages.

### 3.2. Combination with Antibiotics

With the rise of multidrug antibiotic resistance among once-susceptible bacteria, such as *Klebsiella pneumoniae*, multiple antibiotics are often combined to produce an effective therapy [91,92]. With combination therapy, the aim is to produce a synergistic or additive effect which eradicates the infection. Although there are a number of studies which suggest that these antibiotic combinations could select for mutations, allowing for broad-spectrum resistances which can be passed on to susceptible bacteria, the data remains contradictory [91,93,94,95,96]. As such, the use of phages and antibiotics in combination could prove to be beneficial, as there is data to suggest that overall activity of formulations is increased (Table 4) and that the formulation selects against drug-resistant phenotypes [97,98,99,100,101]. This synergistic potential has also been demonstrated for a number of endolysin/antibiotic combinations [102,103]. However, the combination of phages and antibiotics could potentially reduce the overall activity of the phage component in an active treatment, by targeting phage-infected and non-infected cells alike, and potentially interfere with phage replication.

In one such study by Oechslin and colleagues, a 12-phage cocktail (PP1131 at 10^8^ PFU/mL) which targeted *P. aeruginosa* was able to increase the *in vitro* bacterial kill by over 4log_10_ over a 24-h period when combined with 2.5 times the minimum inhibitory concentration (MIC) of ciprofloxacin (a known inducer of prophages) or meropenem when compared to phage or antibiotic alone. *In vivo* studies by the same group showed that the cocktail possessed a half-life of approximately 2.3 h in the plasma while the half-life was reported as approximately 9 h in other organs and that in animals with endocarditis, negative cultures (≤2log_10_ CFU/g) were observed in seven of the 11 animals provided combination therapy compared to monotherapy groups (≥6log_10_ CFU/g) [80].

The combination of antibiotics and bacteriophage-derived endolysins has also been shown to increase antibacterial activity components in Gram-positive bacteria. A study by Becker et al. demonstrated that there was a strong synergy between the LysK and lysostaphin using a turbidity reduction assay providing a calculated ΣFIC of 0.45 ± 0.07 [102]. A synergistic effect was also shown by Garcia and colleagues who demonstrated that the combination of the Staphylococcal endolysin LysH5 with Nisin was able to induce synergy and increase the antibacterial activity eight-fold against a single strain of *Staphylococcus aureus* in a checkerboard MIC assay and pasteurized milk [104]. While this approach shows promise for Gram-positive pathogens, there is limited knowledge on the combinations of endolysins and antibiotics against Gram-negative pathogens.

The global consumption of antibiotics has increased between 2000 and 2010 and there are a number of different strategies for combatting this. The introduction of antibiotic stewardship programs is one such strategy to reduce consumption in human and veterinary medicine [2,105,106,107]. Although such stewardship programs and combinations are shown to possess advantages (increased overall activity, etc.) combining phages with antibiotics is counterintuitive, if the overall aim of these programs is to reduce the consumption of antibiotics. Such a move would not necessarily significantly impact the amount of antibiotics used in combinatorial therapy and the need for broad-spectrum agents would also not decrease. As such, additional research into combinatorial therapy with other non-antibiotic agents should be undertaken.

### 3.3. Combination with Natural Products with Antibacterial Activity

Although much focus has been placed on the combination of phages with antibiotics (both bactericidal and bacteriostatic), there is little in the way of investigation into the effects of non-antibiotic antibacterial compounds in combination with phages (Table 5). As with the development of phage therapy, the use of bulk natural compounds or chemical extracts of these compounds continues to attract interest as alternative antimicrobial agents as both solo agents and also as antibiotic adjuncts [108,109]. The induction of a non-phage-based synergistic or additive response represents an alternative way to increase the overall level of phage therapeutics while potentially simplifying the dynamics of the therapy. However, while research into combination therapy with antibiotics is ongoing, the synergistic potential of phages with other non-antibiotic antibacterial adjunct compounds is a currently-understudied area [97,98,99,110,111].

As with phages, honey has a long history as an antimicrobial agent and has been shown to target multiple bacterial pathogens, inhibit biofilm formation, and may increase wound healing [127,128,129,130,131]. The broad antimicrobial properties of honey stem from the presence of hydrogen peroxide [132], a high sugar content, and the presence of methylglyoxal (MGO) and the antimicrobial peptide bee defensin-1 [133]. However, it should also be noted that the composition of antimicrobial agents between honeys differs dramatically even within localized areas [134,135]. A recent study by Oliveira and colleagues has also shown that phages in combination with one of two Portuguese honeys were able to increase the bactericidal effect against *Escherichia coli* biofilms over a 24 h period compared to the phage or alone. However, this increase in antibacterial activity was coupled with a reduction in phage titer of at least 1log_10_ within 60 min compared to the inoculum at the lowest concentration of honey tested [60]. This broad-spectrum antimicrobial activity is undoubtedly useful, but would present a potential hazard if trying to combine honey with endolysins as both MGO and hydrogen peroxide act against protein structures via cross-linkage or hydroxyl radical formulation, respectively [136].

The risk of broad spectrum antimicrobial activity when combined with bulk antimicrobial plant extracts is also seen in Pimchan et al [59]. This study compared the antibacterial effects of three different bulk plant extracts from plants with known antibacterial properties in combination with two different *E. coli* phages. As the plant extracts were diluted to reduce the potential adverse effects on the viability of the phages, a loss of phage titer of approximately 1log_10_ was only observed for one of the conditions tested. However, this also reduced the overall antibacterial activity compared to the phage alone [59].

Volatile essential oils, such as thymol and carvacrol, extracted from plant materials have also been previously shown to possess potent antimicrobial properties [137,138]. A study by Ghosh et al. evaluated the antibacterial properties of Staphylococcal Phage K and a number of essential oil compounds on the *in vitro* growth of *S. aureus*. While both essential oils tested individually were able to significantly reduce the growth of multiple strains of *S. aureus* at 37 °C, when combined, the effect was not greatly changed [139]. In contrast to this, Chang and colleagues demonstrated synergy between carvacrol and the LysSA97 endolysin in milk with individual components producing approximately a 1log_10_ reduction in bacterial content while the combination reduced bacterial content by approximately 4.5log_10_. This study also suggested that an increased lipid content decreased the synergistic activity [123].

While the combination of natural products and bacteriophages appears to exhibit some promise, extra attention should be paid to the characterization of active components within bulk compounds. Such an approach could potentially yield compounds with potent and specific antibacterial properties. In addition, determining the mechanism of action of such compounds would also be of use. In particular, the selection of compounds which do not interfere with protein structure would be advantageous for both whole phage and phage-derived antimicrobial enzymes.

### 3.4. Combination with Non-Antibacterial Compounds

Although the combination of additional antibacterial agents with phages or their derived antimicrobial enzymes could increase the overall activity of formulations and would be advantageous in intervention therapies, it only enhances a single aspect, the overall kill, and only targets vegetative bacterial species. For preventative measures the overall level of bacterial kill is less important and the ability of the phage to infect its bacterial host becomes more important. As such, the enhancement of the binding and adsorption efficacy of the phage or phage-derived antimicrobial enzyme (e.g., endolysins) could also increase the efficacy of preventative treatments.

The presence of divalent ions (particularly Ca^2+^ ions) is well known to play a role in enhancing the binding efficacy of phages and many phage buffers consequently are supplemented with Ca^2+^ ions (Table 5) [120,140,141,142]. A study by Bandara and colleagues showed that Ca^2+^, Mg^2+^ or Mn^2+^ ions were required to allow *Bacillus* Phages BCP1-1 and BCP8-2 to infect *Bacillus cereus* strains in a fermented soybean paste [143]. A study by Garcia and colleagues combined the Staphylococcal endolysin LysH5 with nisin, in the presence of metal ions. They concluded that LysH5 activity was enhanced in the presence of Ca^2+^, Mg^2+^ and NaCl but inhibited by Mn^2+^ and Zn^2+^ [104]. Therefore, the inclusion of ion chelators, such as ethylenediaminetetraacetic acid (EDTA), as excipients could potentially decrease the efficacy of phage or endolysin binding, while an ionic solvent with increased Ca^2+^ ion concentration could potentially increase the overall level of phage binding [67,144].

### 3.5. Formulation Against Spores and Biofilms

When presented with unfavorable conditions, such as depleted nutrient sources, and the presence of antimicrobials, some bacterial species have developed strategies to enable them to persist in the environment. These include the ability to form bacterial endospores as is the case with *Bacillus* and *Clostridia* species and the ability to produce bacterial biofilms which are considered more resistant to antibiotic and chemical decontamination treatments compared to their vegetative counterparts [145,146]. Phages and endolysins from these two bacterial species have been reviewed elsewhere in more detail [147].

Bacterial spores could be targeted with germinant compounds in order to convert the potentially-resistant spore form into the susceptible vegetative state [148], while extracellular capsules which prevent phage infections could be degraded through the addition of recombinant enzymes [17,149], although there is some suggestion that endolysins may be capable of interacting with the endospore [150]. However, it is important to note that the induction of spore germination in human therapeutics would be highly dangerous and unethical, but for non-clinical applications (particularly wide-area decontamination), the induction of spore germination has resulted in enhanced bacterial destruction [146,151]. The type of germinant that would be required would vary between bacterial species, with bile salts commonly used for *Clostridia* species and a combination of l-alanine and inosine effective in *Bacillus* [148,152,153,154]. However, additional research into the efficacy and interactions between phages or phage derived antimicrobial enzymes is still required.

Bacterial biofilms represent a serious healthcare issue accounting for up to 80% of all microbial infections and are often prevalent in a number of chronic conditions. Such conditions, including cystic fibrosis lung infections and chronic burns, are often poly-microbial in nature [155,156,157]. In these conditions, the biofilm acts in a variety of different ways which can result in the failure of antibiotic treatment [158]. Biofilms can act as a permeability barrier to both antibiotics and antimicrobial metals, as well as to non-specific immune mechanisms [159,160,161,162]. While phages are capable of reaching bacteria within biofilms, the production of exopolymeric substances (EPS) can act as a non-specific barrier to phages, reducing the amount that are able to infect susceptible cells. In response to this, some phages possess EPS de-polymerases which can disrupt biofilms (Table 6). Although these enzymes are not directly antimicrobial, they could potentially increase the amount of phages that can reach bacterial targets by degrading EPS, while disrupting the overall biofilm structure and exposing persister cells [163,164,165]. The development of phage derived EPS de-polymerases may initially be more inefficient due to the costs associated with research and regulatory approval and the use of traditional approved mucolytic agents such as ambroxol or acetylcysteine may prove more financially viable [166,167].

## 4. Enhancing Storage, Dosing, and Delivery

Any final formulation involving whole phages, phage-derived antimicrobial enzymes, and adjunct components will have to consider the intended application. How such therapeutics are stored and delivered could influence the potential shelf life, persistence *in vivo*, and the overall efficacy of the phage and non-phage adjuncts [13,181,182]. Although clinical and non-clinical applications share some of the same requirements for storage and delivery (e.g., long shelf life at ambient temperatures), which would enable research to be carried out more effectively, it is likely that each treatment area will possess unique requirements. 

The susceptibility of whole phages to environmental stresses (pH, temperature, humidity, etc.) can be highly variable under non-formulated conditions, although most can tolerate relatively wide pH and temperature ranges [14,126,183,184,185,186]. These varying degrees of susceptibility will have an important impact, not only during manufacturing, formulation, and routine storage, but also during therapeutic applications, particularly when encountering a hostile environment (e.g., transiting the stomach) [186]. In order to increase the long-term storage of phages, they have been lyophilized in the presence of different sugars for increased stability and allowing delivery in a powdered form, e.g., in metered-dose inhalers [187,188,189]. Additionally, phages or their derived antimicrobial enzymes could be formulated into liquid formulations, but in order to maintain pharmacopeia compliance, preservatives or other compounds may be required to prevent microbial contamination.

As discussed earlier, the overall efficacy of a phage cocktail could be compromised (assuming an immediate application of all phages) as one phage reproduces more efficiently than the other components (e.g., more efficient adsorption and shorter latency period) or as phages compete for binding sites when applied simultaneously. This competition could be reduced by the selection of phages which do not compete with one another in cocktails or applying individual phages at different times initially, as there are also solutions through formulation which could enhance and simplify this process. Modified release dosage allows for drug delivery with a delay from the time of administration or prolonged release and is used for pain management [190,191,192]. Simple liquid suspensions are most commonly used for topical and oral applications [193,194,195,196]. While these suspensions are able to effectively rinse infected wounds as large volumes of suspension can be applied, much of the suspension is lost as it runs off the site of infection. However, for applications which require a surface contact (e.g., food preservation and wound infections), impregnated materials, such as hydrogels (Table 7), are increasingly being researched as these would allow for a constant release of phage or antimicrobial enzyme by maintaining contact with the contaminated area. This could ultimately result in less wastage, but optimization would be required to ensure that an effective dose is delivered.

Encapsulating the phage or derived antimicrobial enzyme within polymer microparticles (Table 7) [177] could also prove beneficial. By encapsulation of biofilm-disrupting compounds, such as ambroxol, which are released prior to the release of the phage, the overall efficacy could be enhanced as they would enable greater penetration into the biofilm layer. Such polymer microparticles have been shown to be able to be triggered into releasing their “drug” components through a variety of triggers, including pH and temperature, each of which would benefit specific applications (e.g., low temperature release for food preservation and low pH to survive transit through the gastrointestinal tract) [179,180]. Encapsulation of formulations within microfluidic-produced microcapsules would also allow for the production of uniformly-sized particles [179,197], which for inhalational use would mean that specific areas of the lung could be targeted [198]. While for larger scale spray applications (e.g., crop protection), uniformly-sized particles in combination with effective delivery technology could optimize surface coverage [199].

## 5. Phage Degradation and Immunogenicity

There is evidence to suggest that the release of bacterial endotoxins (in particular lipopolysaccharide; LPS) does not increase and that the overall outcome of phage therapy is not compromised due to anti-phage immune responses in phage-treated complex systems [20,137,200,201,202]. There are still areas in which further research is necessary, as the level and type of immune response encountered is likely to change depending on the therapy approach. For example as an additive to animal feed, where phages would be given over long periods of time, this could result in the development of phage-specific antibodies [35,203]. While the incorporation of immunomodulatory compounds into therapies could be potentially ethically and practically inadvisable, there are a number of anti-LPS compounds (such as lipoamines or some alkaloids) that have been shown to reduce pro-inflammatory responses caused by LPS [204,205,206,207]. However, these would need to be tested to determine their ability to work in synergy with phages or their derived antimicrobial enzymes.

An alternative approach would be to mask phages or antimicrobial enzymes from the immune system. Such an approach has been described by Kim et al. This approach involved the pegylation of whole phages and suggest that phage half-life in a mice was increased and exhibited significantly lower levels of IFN-γ and IL-6 release [208,209]. However in the case of endolysins, a later study by Resch et al. showed that the pegylation of a *Streptococcus pneumoniae* specific endolysin (Cpl-1) exhibited significantly lower activity than that of the native enzyme [210].

Within reported human cases within Western hospitals, dosing requirements are often based on the endotoxin content of a phage preparation rather than the phage content in order to maximize the amount of phages that can be delivered during a single treatment while meeting regulatory requirements [87,202]. It should be noted that some phage clinical trials have provided little information about the overall dosing and concentration of the active phage component [211]. While such studies have shown promise, there is still limited knowledge surrounding the dosing requirements for phages and phage-derived antimicrobial enzymes when applied *in vivo* as antibiotic alternatives.

## 6. Concluding Remarks

Therapeutics based on whole phages (or their derived antimicrobial proteins) represent an exciting alternative to conventional antibacterial treatments in both clinical and non-clinical scenarios. While for human clinical applications, there are a number of clinical successes reportedly supported by growing *in vitro* testing and *in vivo* testing which suggests that whole-phage therapy is viable for a variety of conditions. However, it is important to note that these reported human cases are often on a limited number of patients and have been based on compassionate or magisterial phage usage rather than taking the form of regulated clinical trials [15,212,213]. In non-clinical applications, whole phages and endolysins have found some commercial success as food additives to prevent bacterial contamination (Table 1), but phage-based medicines have yet to successfully navigate the clinical approvals process. For whole phages this partly stems from an incompatibility with regulatory processes and the potential need for phage-specific approval pathways. It also reflects a need for increased collaboration between regulators and developers to decide what form phage therapy should take (bespoke or pre-manufactured), the desired outcome of any treatment (prevention or intervention) and the type of regulation that is necessary for widespread implementation (full regulatory approval or compassionate usage only) as for each area different selection criteria could be required.

Currently available commercial products or compassionately-used therapies often contain simple cocktails of phages or phage-derived antimicrobial enzymes (e.g., endolysins) that are, in the majority of cases, applied directly to target areas and contain few, if any, additional components. Indeed, while the development of more complex pharmaceuticals with phages or phage-derived antimicrobial enzymes as their main active ingredients may allow for some of the limitations to be addressed, this is currently understudied. However, it would be necessary to study the pharmacology and secondary effects of such combinations *in vitro*.

It is unlikely that whole-phage products will become the widespread antibiotic alternative that they are considered to be by some. While the implementation of complex formulations may help to address some of the biologically-imposed limitations of whole-phage treatments, these will come with additional financial costs and also further complicate the already complex nature of phage pharmacology. While the information presented in the current review is not an exhaustive comparison, it is important to note that any complete formulation will likely be the result of tradeoffs designed to maximize benefits and minimize deficiencies.

## Figures and Tables

**Table 1:** Summary of commercially available whole phage and phage-derived antimicrobial enzyme. FDA: Food and Drug Administration, USA, USDA FSIS: United States Department of Agriculture Food Safety and Inspection Service, GRAS: Generally Recognized as Safe, EPA: Environmental Protection Agency, USA.

	Manufacturer	Product Name	Application	Approval Status	Reference
Whole Phage	Intralytix	ListShieldTM	Targets *Listeria monocytogenes* in food/food processing	Complies with FDA food additive rules	[37]
				USDA FSIS listed safe	
				EPA-registered	
				Health Canada approved	
				National Food Services of Israel approved	
		EcoShieldTM	Targets *E. coli* 0157:H7 in food/food processing	FDA Cleared	[38]
				Health Canada approved	
				National Food Service of Israel approved	
				USDA FSIS listed safe	
		SalmoFreshTM	Targets highly pathogenic Salmonella-serotypes in food/food processing	USDA FSIS listed safe	[39]
				GRAS for direct application	
				Health Canada approved	
				National Food Service of Israel approved	
		ShigaShieldTM	Targets Shigella species in food/food processing	GRAS for direct application	[40]
	OmniLytics	AgriPhageTM	Targets bacterial spot, bacterial speck and bacterial canker on tomato and pepper plants	EPA registered	[41]
	Pherecydes Pharma	PhagoBurn	Targets skin infections in burn wounds	Phase 2 clinical trials	[42,43]
		PneumoPhage	Targets *Pseudomonas aeruginosa* in acute respiratory tract infections	-	[44]
		Phosa	Targets *Staphylococcus aureus* and epidermidis in bone infections	-	[45]
	AmpliPhi Biosciences Corporation	AB-SA01	Targets *S. aureus*	Expanded Access	[46]
				Phase 1 completed	
		AB-PA01	Targets *Pseudomonas aeruginosa*	Expanded Access	
Endolysin	Micreos Human Health	StaphefektTM	Endolysin that targets *S. aureus* and MRSA	Interventional Clinical Trial	[15,47,48]
	ContraFect	CF-301	Phage-derived lysin that targets *S. aureus* blood stream infections	Completed Phase 1 clinical trials	[49]
				Granted Fast Track Designation from FDA	

**Table 2:** Requirements for pharmaceutical preparations by application type. G^−^: Gram-negative [13,68,69,70,71].

Route of Administration		Total Aerobic Microbial Count (CFU/g or CFU/mL)	Total Combined Yeast/Mold Count (CFU/g or CFU/mL)	Absence of Specific Microorganisms	cGMP Requirement	Defined Endotoxin Limits
Oral	Non-aqueous	10^3^	10^2^	*E. coli*	Yes	No
	Aqueous	10^2^	10^1^			
	Buccal/Gingival	10^2^	10^1^	*S. aureus*		
Skin	Transdermal	10^2^	10^1^	*S. aureus* and *P. aeruginosa*	Yes	No
	Cutaneous					
	Injectable	0	0	-		Yes
Vaginal		10^2^	10^1^	*S. aureus* and *P. aeruginosa* and *Candida albicans*	Yes	No
Rectal		10^3^	10^2^	-	Yes	No
Inhalation		10^2^	10^1^	*S. aureus, P. aeruginosa* and bile tolerant G^−^ bacteria	Yes	No

**Table 3:** Summary of non-safety *in vivo* phage cocktail studies. i.c.: intracavity wash; i.v.: intravenous.

Condition Targeted	Bacterial Species	Cocktail Composition	Results	References
Human Chronic otitis	*P. aeruginosa*	Biophage PA (six phages; BC-BP-01 to BC-BP-06) 10^5^ PFU per phage	Clinical indicators improved in phage treated patients compared to placebo *P. aeruginosa* counts significantly lower in phage treated group compared to placebo	[86]
Human necrotic pancreatitis	*Acinetobacter baumnnii*	Three phage cocktails used; φPC (i.c.), φIV (5 × 10^9^ PFU i.v.) and φIVB (5 × 10^9^ PFU i.v.)	Patient survived and fully recovered Resistance to cocktails φPC and φIV after 8 days Phage serum concentration dropped to 20 PFU/mL after 120 min	[87]
Murine Bacteremia	*Klebsiella pneumoniae*	GH-K1, GH-K2, GH-K3	Cocktail reduced bacterial titre approx. 3–4log_10_ compared to monophage Cocktail counts decreased ≤2log_10_ in 90–120 min	[88]
Necrotic Enteritis of boiler chickens	*Clostridium perfringens*	*C. perfringens* phages (CPAS-7, CPAS-12, CPAS-15, CPAS-16and CPLV-42) in equal amounts	Mortality <1% when administered with water or feed. Compared to 64% in controls	[89]
Mouse model of mastitis	*S. aureus*	Twelve phage cocktail (composition unknown except for two phages; BP39 and mutant of ATCC 23361)	Bacterial counts approximately 4–5log_10_ in mammary tissue for cocktail treated group compared ≤2log_10_ for cefalonium treated control.	[90]

**Table 4:** Examples of phage or derived antimicrobial enzymes and antibiotic combinations. Antibiotic abbreviations based on BSAC where available [112].

	Bacterial Target	Combination Tested	Results	References
**Phage**	*P. aeruginosa*	Phages σ, σ-1 or 001A/subinhibitory GEN, CIP, ceftriaxone or polymixin B	No additive effect in GEN and polymixin B combinations Ceftriaxone combinations showed ≥2log_10_ reduction compared to individual components by 300 min	[98]
		Phage LU27/Streptomycin 120 or 240 µg/mL	Phage only control showed approx. 1log10 reduction at 70 h Bacterial reduction 2–3log_10_ reduction in 100 µg/mL streptomycin/phage combination compared to streptomycin only at 70 h Delay in antibiotic addition altered pattern of kill	[113]
	*Burkholderia cepacia* Complex	Phage KS12/1.25 µg/mL CIP, 5 µg/mL MEM, 5.5 µg/mL TET	Phage/CIP and MEM combinations showed ≥3log_10_ reduction at 325 min compared to controls	[99]
	*E. coli*	Phage φMFP/50 or 20 ng/mL CTX	Phage titer increased by approx. 1log_10_ by 120 min post administration Plaque sizes increased compared to control	[114]
	*K. pnuemoniae*	Phage B5055/CIP	Reduction of bacterial biofilm content of approx. 5log_10_ 180 min after phage addition No significant difference in reduction between combination and phage only treated biofilms Frequency of resistant variants decreased in combined testing compared to individual components	[115]
	*S. aureus*	Phage MR-10/5 mg/Kg MUP	Phage/MUP combination showed >1log_10_ reduction in bacterial content by day 3 post treatment in BALB/c mice compared to controls Clearance in Phage/MUP combination by day 5 Individual components showed clearance by day 10	[116]
**Derived antimicrobial enzymes**	*Streptococcus pneumoniae*	Endolysin Cpl-1/GEN or PEN	Synergy with PEN or GEN was dependent on level of PEN resistance	[117]
	*A. baumannii*	Endolyin LysABP-01 (concentrations ranged from 0.0156-2 × MIC)/CIP, IMP, COL, CHLO, GEN, ERY, or TET (concentrations ranged from 0.0625-2 × MIC)	Phage/COL showed elevated antibacterial activity (close to 100%) in comparison to other phage/antibiotic combination	[118]
	*Listeria monocytogenes*	Endolysin PlyP100/nisin	Endolysin stable for up to 28 days under cold storage PlyP100/nisin combination maintained activity over 4 weeks	[119]
	*S. aureus*	CF-301/ DAP, VAN	Synergy between CF-301 and DAP/VAN resulted in increased murine survival compared to when just treated with antibiotics alone (*p* < 0.0001)	[103]

**Table 5:** Examples of phages or derived antimicrobial enzymes in combination non-antibiotic compounds.

	Bacterial Target	Combination	Results	References
**Phage**	*K. pneumoniae*	Phage KPO1K2 or NDP/CoSO_4_ or FeCl_3_	Reduction of ≤1log_10_ in NDP/10 µM FeCl_3_ combination in biofilms up to seven days versus untreated control Reduction of 1–2log_10_ in KPO1K2/10 µM FeCl_3_ combination in biofilms up to seven days versus untreated control Reduction of ≥5log_10_ in KPO1K2/10 µM FeCl_3_ + 500 µM CoSO_4_ combination in three day old biofilms versus untreated control	[120]
	*E. coli* O157:H7	Phage cocktail BEC8 (10^6^ PFU/leaf)/0.5% *v*/*v trans-*cinnameldehyde	Total kill (4–6log_10_ reduction) within 10 min when combined at all conditions Individual treatment results varied based on bacterial inoculum and incubation temperature	[121]
	*L. monocytogenes*	Listex P100/potassium lactate and sodium diacetate	Prevented *L. monocytogenes* outgrowth for up to28 days compared to controls Smaller reduction in bacterial count seen at lower temperatures	[122]
**Derived antimicrobial enzymes**	*S. aureus*	Endolysin LysSA97 (376 nM)/carvacrol (3.33 mM)	Individual components showed reduction in bacterial content of approx. 1log_10_ Combination reduced bacterial content >4log_10_ Combined activity varied depending on lipid content	[123]
	*L. monocytogenes*	Endolysin PlyP825/High hydrostatic pressure (HHP)	Synergistic inactivation of *L. monocytogenes* in milk, cheese, and smoked fish Allows for lower pressure level to be used with the same antimicrobial efficacy when treated in combination with phage Decrease bacteria positive food samples during storage	[124]
		Endolysin PlyP40, Ply511 or PlyP825/High pressure	Individual treatment reduced bacterial number ≤1log_10_ Combined treatment reduced bacterial number ≥5log_10_	[125]
	Various	Endolysin Lys68/weak acids	Lys68/EDTA combination only lysed *Pseudomonas* Lys68/citric or malic acid effected 9 or 11 species, respectively Bacterial reduction <3log_10_ for all conditions tested	[126]

**Table 6:** Examples of bacteriophage-derived depolymerase enzymes.

Bacterial Target	Phage	Summary	Reference
*A. baumannii*	Petty	Enzymatic activity from Dpo1 depolymerase protein from Petty degraded purified EPS from *A. baumannii*	[168]
	ϕAB6	ϕAB6 has a polysaccharide depolymerase degraded *A. baumannii* EPS and is a component of the phage tail fiber that determines host specificity	[169]
*E. coli*	VB_EcoM_ECOO78	Dpo42 prevented biofilm formation in 15 clinical *E. coli* strains and reduced biofilm formation when compared to negative controls	[170]
	K1, K5, and K30	Addition of polymerase increased mouse survival at five days post bacterial exposure in a concentration dependent manner Differences in survival were observed between different depolymerase types Depolymerase in combination with serum enhanced bacterial killing compared to controls	[171]
*Klebsiella*	ϕK64-1	Phage encoded 8 putative depolymerases Production of phage mutants that did not encode putative depolymerases eliminated lytic activity	[172]
	K5-2 and K5-4	Each phage encodes for two different capsule depolymerases that allows them to replicate on certain *Klebsiella* strains K5-2 causes spots on seven capsular types of *Klebsiella* K5-4 increased survival of mice treated with *K. pneumoniae* K5 strain	[173]
	KP32	Tail tubular protein A (TTPA), a structural tail protein of KP32, exhibits lytic activity towards EPS. TTPA can be regarded as a dual function macromolecule with both structural and enzymatic activities	[174]
*Erwinia amylovora*	L1	DpoL1 is required for L1 uptake and specifically binds to, and degrades, *E. amylovora* EPS by cleaving the amylovoran galactose backbone	[175]

**Table 7:** Examples of modified-release dosage of phages or phage-derived antimicrobial enzymes. HPMC: Hydroxypropylmethylcellulose; PNIPAM: Poly (*N*-isopropylacrylamide).

	Bacterial Target	Formulation	Results	References
**Phage**	*K. pneumoniae*	Phage Kpn5/HPMC hydrogel	Enhanced survival (≥60%) of burnt mice over five days	[176]
	*S. aureus*	Phage K/alginate microspheres	Encapsulation significantly improved survival in simulated gastric fluid Incorporation of trehalose, sucrose, skimmed milk, or maltodextrin-improved phage viability following drying	[177]
	*Salmonella* Enteritidis	Phage f3αSE/ alginate spheres	Encapsulation extended phage release to over 200 h compared to control	[178]
	*Propionibacterium acnes*	Phages PAC1-10/cetomacrogol cream	Phage activity maintained over 90 days when preparation maintained at 4 °C in the dark	[51]
	*C. difficilie*	Phage CDKM9/Eudragit ± alginate	Encapsulated phage withstood simulated gastric fluid for 3 h Release triggered at pH 7	[179]
**Derived antimicrobial enzymes**	*S. aureus*	Endolysin CHAPk and lysotaphin in PNIPAM nanoparticles	Both enzymes work synergistically to lyse *S. aureus* with a fast response time in comparison to choice antibiotic used for MRSA treatment PNIPAM allowed for successful diffusion while maintain stability of the enzybiotic cocktail	[180]
